# Attentional Competition and Semantic Integration in Low- and High-Span Readers

**DOI:** 10.3389/fpsyg.2022.871094

**Published:** 2022-05-20

**Authors:** Connie Qun Guan, Scott H. Fraundorf, Mingle Gao, Chong Zhang, Brian MacWhinney

**Affiliations:** ^1^School of Foreign Studies, Beijing Language and Culture University, Beijing, China; ^2^Department of Psychology, Carnegie Mellon University, Pittsburgh, PA, United States; ^3^Department of Educational Psychology and Learning System, Florida State University, Tallahassee, FL, United States; ^4^Department of Psychology, University of Pittsburgh, Pittsburgh, PA, United States; ^5^Learning Research and Development Center, University of Pittsburgh, Pittsburgh, PA, United States; ^6^National Institute for Education Sciences, Beijing, China

**Keywords:** attentional competition, semantic integration, working memory, distractive elaboration, predictive inference, text memory

## Abstract

The goal of the current study is to investigate the effects of the distractive textual information on the activation of predictive inference online, and how the readers with high or low working memory capacity (WMC) differ in their online activation and text memory. To test the two hypothesis of attentional competition (AC) and semantic integration (SI), we conducted three experiments to investigate whether a local prediction (e.g., “The vase broke”) and a global prediction (e.g., “The wife left her husband”), both of which could be derived from the description of a critical event (e.g., “The angry husband throws the delicate porcelain vase against the brick wall”), are generated in the mind of the reader, and how this generation process is influenced by contextual and cognitive factors of the reader (e.g., working memory capacity). The results of Experiment 1 and 2 suggest that the elaboration of the global aspects in the narrative reduces the local prediction, but makes the global prediction more salient to occur. The evidence from Experiment 3 confirms the hypothesis that even automatic processes are constrained by distant contextual factors, in combination with differences in working memory, and examines how referentially local and global predictions are intertwined in text comprehension. Overall, these data support the immediate integration hypothesis across sentence boundaries at different representation levels (cf. Schmalhofer and Perfetti, 2007), as well as interaction assumptions of different processing levels within referentially local and referentially global processing contexts (cf. Yang et al., 2005).

## Introduction

Successful text comprehension requires a well-timed interplay of online processing and offline memory. Readers must quickly draw inferences from the information given in the text and combine them with their preexisting knowledge so that a coherent situation model emerges in the reader’s mind (van Dijk and Kintsch, 1983; Zwaan and Radvansky, 1998). In order to keep an interaction going, readers must adapt their attention to either suppress or construct incoming information with prior world knowledge or text memory. Adult readers manage to accomplish these complex processes with ease, which raises two questions. How does the processing of the local information occur online, and how does this online activation of the local information persist in the long-term text memory. What are the mechanism of online processing and representation of text? Second, how do these online successful information processes build up to create offline memory for text?

A large number of theories have recently converged on the idea that both of these questions can be answered by the notion that readers make predictions online and link them to long-term text memory (van den Broek, 1990; Ericsson and Kintsch, 1995; Rabagliati et al., 2017). Testing this effect on text comprehension is crucial because it reflects the linkage between the moment-to-moment online text processing and the offline text memory (Calvo, 2000; Cook et al., 2001; Linderholm, 2002; Schmalhofer and Perfetti, 2007). Although there is extensive research examining whether readers generate inferences online, less attention has been paid to the connection between online inferences and some subset of offline text memory with which they “resonate” (Myers and O’Brien, 1998) as related information in the text-encoding process.

These theories often distinguish between two types of inferences: local inferences and global inferences. For instance, when readers encode the sentence “The angry husband threw the delicate porcelain vase against the brick wall,” they may thus generate, in combination with prior knowledge about the fragility of porcelain, the inference “The vase broke.” And if some preceding segments of the text had furthermore elaborated that “his wife *Susan* had threatened her husband, *Steven*, that she was going to leave him if there was even the mildest violent incident in the house,” readers could furthermore generate the inference “Susan left her husband.” Both of these inferences have been termed predictive inferences (McKoon and Ratcliff, 1986; Graesser et al., 1994). They differ, however, with respect to their scope of reference. The first inference has a relatively small scope that focuses on a particular object, namely the vase and its physical appearance at a particular point in time; according to the earlier literature, such inferences are therefore referentially local or, in short, *local predictions*. Meanwhile, the inference about “Susan’s leaving” her husband addresses the top-level topic of the story about the relation between a wife and her husband; we term such inferences *global predictions*. However, research so far has not been explored the degree to which these two types of inference occur online as function of divided attention or text integration, nor how they are retained in long term memory.

In the current study, we explore the relationship between local and global inferences and the textual conditions under which they are activated, both online and offline. We use both a word-stem completion task and word-probe naming task to achieve these goals. Based on the debate between the minimalist hypothesis (McKoon and Ratcliff, 1992) and Construction-Integration hypotheses (Kintsch, 1994) as to the degree to which these inferences occur online, we examine individual differences in terms of high and low-span readers in language and cognitive processes during reading and after reading. In doing so, we provide a novel examination of the link between online activation ad offline long-term text memory integration so that we can better determine whether and to what extent the online competition of the text construction. There are two separate possibilities. It’s online competition and integrative processes that could play a role in readers with varied memory capacity.

### Predictive Inferences in Reading Comprehension

*Predictive inferences* are conclusions about an event (e.g., the vase breaking) that occur when contextual information has become so constrained that an event is very likely to have occurred (Keefe and McDaniel, 1993). Experimental evidence for predictive inferences has often come from naming tasks; when a target word to be named is related to an inference (e.g., the word “broke”), it is produced more quickly, suggesting that some kind of inferencing or inference-related processing must have occurred. Through a large variety of controls, it is also known that such speeded pronunciation is not simply due to contextual factors, but indeed indicative of specifically heeded information or similarly focused attention (e.g., Ericsson and Simon, 1993), that is more or less closely related to the contents of the targeted inference “The vase broke.”

Much experimental work has established that inferences are made during reading to maintain local coherence (McKoon and Ratcliff, 1986), to establish causal relations within a text (van den Broek, 1990), and to establish the meaning of a piece of discourse (Graesser et al., 1994). Research shows that inferences take time to develop (Calvo and Castillo, 1996, 1998) and that inferences are retained for later retrieval in a *situation model* (Durgunoglu and Jehng, 1991; Klin et al., 1999; Guan, 2007). In short, the core purpose of making inferences is to make implicit connections explicit and link the current textual information with earlier text as well as with the reader’s world knowledge. This is considered “a prerequisite for learning” (Kintsch, 1994, p. 302).

Although there is consensus that predictive inferences are critical to comprehension in at least some instances, there has been controversy on how frequently they are drawn. Kintsch and Keenan (1973) suggested that readers infer the respective proposition (e.g., the vase broke) in the same way as other propositions that were presented explicitly in the text. But according to McKoon and Ratcliff (1992) *minimalist hypothesis*, inferences are drawn only when they are either needed to maintain local coherence of the text or when they are easily available (Keefe and McDaniel, 1993; Fincher-Kiefer, 1995; McDaniel et al., 2001). Supporting this, predictive inferences have been shown to occur mostly frequently when the preceding context is very constraining and supports only one inference (Murray et al., 1993; Cook et al., 2001).

Previous research evaluating these hypotheses has focused primarily on the methodological differences or on a single text variable, such as the role of predictability of the crucial target event. Given the theory of inferencing, predictive inferences should be more likely to drawn if they are highly constrained by the context, and if there are few alternative consequences that can occur (Fincher-Kiefer, 1995). However, multiple inference possibilities could also occur depending on the varied levels of alternative consequences of readers’ heeded information. In this current study, we test this possibility by focusing on the global and local scope of referential features of the multiple activation scenarios (i.e., local prediction and global prediction). We also examine the exact content and the representational nature of this heeded information, which is not yet well understood.

### The Assumption of Attentional Competition

The local on-line predictions which a reader makes during reading can vary in their specificity. The informational content of a minimal inference is a sort of emotional qualification of the upcoming event, such as “something bad happened,” but not a distinct propositional representation of the event, such as “The vase broke.” But, inferences can become more specific; for instance, Gueraud et al. (2008) showed that by adding supplementary information (e.g., it was a birthday party) to a preceding text that described a dinner party, some heeded information (“dessert was served”) becomes more specific (“cake was served”). Their results showed that, during the comprehension of a text, there was indeed a continuous attentional competition (AC) between a more general or a more specific prediction of the event which may happen next. This general prediction is what we called global inference, and the specific prediction is what we called local inference, in details, the predictive inference induced by the target sentence without the contextually elaborated text. The different levels of activation of both global and local inference could makes this a competition.

When the contextual support for a prediction is relatively weak, that context activates several likely events in general world knowledge that could follow, and the heeded information could possibly be split among these possible events at a conscious or subconscious level (e.g., Dehaene and Naccache, 2001). But when contextual support increases for a specific inference, the heeded information changes, and a single event consistent with the context will receive a higher level of activation as a function of a newly read proposition, processing strategy, and memory capacity. These involve two competing hypotheses: attentional competition between the two types of inferences, and the immediate semantically text integration between these two types of inference. How the heeded information changes in this manner over several processing cycles has been spelled out computationally by the associative network model of Schmalhofer et al. (2002).

### The Assumption of Immediate Semantic Integration

Perfetti and associates have suggested that reading comprehension and inferencing (Schmalhofer and Perfetti, 2007) is best conceptualized as a sequential word-by-word process in which the words of a sentence and various cognitive units (lexical, conceptual, and situational items) are attended to and subjectively related in the reader’s mind. According to this account, a reader’s local and global predictions (e.g., the vase broke and Susan left) form not a minimal but an optimal cognitive preparation for the possible ways that two sentences might be semantically integrated. This is an opposing hypothesis to the minimalist view of inference activation in which the degree of predictive inference activation is at the lowest level. More specifically, Yang et al. (2005) showed in an ERP study that such inferential integration processes occur immediately; that is, at the earliest possible processing stage. Depending on the specific characteristics of the reader and the text, SI may thus occur at a lexical, conceptual, or situational level.

The time course of SI appears to depend on an individual’s comprehension skill, as suggested by ERP studies by Yang et al. (2005) and Perfetti et al. (2008). Their results showed that skill differences were represented by the readers’ ability to use different levels of linguistic information effectively in context-appropriate retrieval and in the selection of word meanings for immediate processing. High-skilled comprehenders have a coherent lexical knowledge structure that allows them to achieve an early SI at the lexical level after 300 ms (Perfetti et al., 2008; P300 for the paraphrase condition) and thereby achieve a refined word-to-referent integration (Perfetti and Hart, 2002). By contrast, less-skilled comprehenders have inefficient lexical processing skills, which passes the burden of SI of the sentence up to the next processing level. Thus, less skilled readers can therefore achieve the integration only at the situational level, after 400 ms (Perfetti et al., 2008; **Figure 1**; early N400 for the paraphrase condition). For referentially-paraphrased sentences, this leads to a delayed and less refined word-to-referent integration. These findings show that SI and inferencing differs between less- and more-skilled readers and that a reader’s working memory (Unsworth and Engel, 2007) may be an important factor in inferencing.

### The Current Study

In the current study, we aimed to assess (a) the online activation status of both local and global predictions (Experiments 1 and 2), (b) whether high- and low-span readers differ in this attentional split or sustained immediate integration of both local and global predictions (Experiment 3A), and (c) how online predictions affect later offline text memory (Experiment 3B).

In Experiments 1 and 2, we examined the conditions under which both referentially local and global predictions are drawn online. We examine whether and to what extent online inferences can be activated as a function of distant context, and which mechanism—attentional competition or SI—explains these patterns. In Experiment 1, we investigate these questions using a word-stem completion task to detect the online activation of both referential concepts. However, a disadvantage of the word-stem task is that it follows after the text; if the text following the inferences does not keep the inferred concept in focus, it may be maintained at an insufficient level of activation to influence naming or recognition latencies. So, in Experiment 2, we used a naming task that was sensitive to immediate activation to examine the degree to which the referentially local concepts were activated online when the global aspect in the narrative was elaborated at a low or a high degree.

In Experiment 3A, we then investigate whether there are individual differences [e.g., as a function of working memory capacity (WMC)] in drawing inference, with the particular aim of addressing how the SI deficits of low-span readers affect varied levels of text representation.

Lastly, in Experiment 3B, we focused on the linkage between local and global predictions made online and later offline text comprehension. Specifically, we do not fully know the extent to which predictive inferences are maintained and encoded in long-term text memory.

The test paradigm in this current study is also feasible as demonstrated by the previous literature (Perarchii and O’Brien, 2004), since no matter whether the information is relevant or not to the understanding of the text, all information that shares either contextual or semantic features can be instantiated by the inferential-probe word and the text recall.

## Experiment 1

Experiment 1 was designed to determine whether—and to what extent—an elaboration on the global aspects in the narrative would affect referentially local predictions. There were three predictions in Experiment 1. *First*, if predictive inferences are processed automatically (McDaniel et al., 2001), the referentially local prediction should be activated regardless of the degree of elaboration on the global aspects in the narrative. *Second*, if the distant context also matters to local predictions, that would suggest that the activation of the local prediction depends on the degree of elaboration supported by the context. *Third*, there may be an interaction effect between the automatic processing effect and the distant contextual effect such that the global prediction can only be activated in high-elaboration contexts but not low-elaboration contexts.

To detect the activation of both local and global predictions, we used a Constrained Word-Stem Completion (CWS) task in which participants must select and name a word that fits the constraints (e.g., *st* as the word stem for the target word *sting*). Prior work has shown that the CWS task is sensitive to predictive inferences: Subjects complete constrained stems with the target inference-related words more often after presentation of contexts that prime those inference than they did after presentation of control contexts (Whitney et al., 1992).

The CWS task has several advantages. It has no binary decision (Forster, 1979) but could be multiple possibilities, since the inference activation could be spurious or multiple online and so hard to detect. Second, the CWS task does not involve relating the target word back to the priming context (McKoon and Ratcliff, 1989). The selection of a particular word candidate may be biased by prior semantic activation, but a post access context check would not be particularly helpful in making the required response (as long as filler trials are used to avoid having the subjects adopt guessing strategies). Lastly, an important advantage of the CWS task over naming and lexical decision is that the processing of the target in the CWS task is not so automatic and data driven as to override the effects of prior semantic activation.

We predicted that this task would providence evidence of global inferences, but the degree of elaboration on the global aspects in the narrative would affect the automaticity of the readers’ responses.

### Materials and Methods

#### Participants

Graduate and undergraduate students enrolled in a large Southeastern U.S. university participated in this study (*N* = 29) in exchange for bonus credit in their courses. One participant’s data were discarded from the final analyses because the accuracy rate for his comprehension questions was below 90%.

#### Materials

There were 24 experimental passages (see Table 1) and 8 filler passages. Each experimental passage contained either a high or low amount of elaboration on the global aspect in the narrative. The high-elaboration context contained on average four sentences ranging between three and six sentences, while in the low-elaboration context, the amount of elaboration on the global prediction was held constant at one sentence. The total number of sentences in both high- and low- elaboration conditions was the same. The elaboration on the global aspect of narrative was followed by two sentences of backgrounding information. Lastly, the final sentence was either an inference-evoking target sentence or a control. Thus, each passage appeared in four different versions: High Elaboration Inference, High Elaboration Control, Low Elaboration Inference, Low Elaboration Control.

**TABLE 1:** Sample text in experiments 1, 2, and 3.

High elaboration condition:
S0: (Cued) Steven and Susan had been married for 20 years.
S1: After years of abuse, Susan had enough.
S2: She joined a support group for battered women and told her husband, Steven, that she was going to *leave* him if there was even the mildest violent incident in the house. S3: Steven was taking her seriously. He had managed to control his temper for the past month.
S4: He couldn’t bear the thought of her *leaving*. He felt his life would be over if she *left*.
S5: Today Susan had left a mess in the kitchen which had enraged Steve.
S6: He felt himself losing it.
Low elaboration condition
S0: (Cued) Steven and Susan had been married for 20 years.
S1: After years of abuse, Susan told Steven she would *leave* him if there was even the mildest violent incident in the house.
S2: In addition, Steven had just started a new job as the assistant manager of the accounting department at Sears.
S3: It meant a lot of extra responsibilities, long hours, and more stress.
S4: Steven and Susan were having a hard time adjusting their life to fit his schedule.
S5: Today Susan had left a mess in the kitchen which had enraged Steven.
S6: He felt himself losing it.
Inferencing-evoking target sentence:
Unable to control his anger, Steven threw a delicate porcelain vase against the brick wall.
Control sentence:
Working hard to control his anger, Steven apologized and offered to clean her delicate vase.
Word stem completion task (Experiment 1 only)
B (the local prediction) and L (the global prediction)
Probe (Experiment 2 only)
BROKE.
Comprehension question
*Did Susan leave a big mess in the kitchen? (Correct answer: Yes).*

The eight filler passages were of the same length as the experimental passages. All of the probe words for the filler items were taken from the filler passage itself. The filler passages were presented before each of four reading versions so as to avoid carry-over effects, which did not include any predictive prompt or elaboration. A yes/no comprehension question was presented at the end of each passage to make sure the participants read carefully.

For each passage, we created constrained word stems (CWS) using the rule in Whitney and Williams-Whitney (1990) study: One letter was provided for words with four or fewer letters, and two letters were provided for words with five or more letters. When providing the first two letters yielded identical stems for different targets (e.g., *sting* and *study*), three letters for the CWS targets were given. A pilot study on 20 subjects showed that all of the targets had baseline completion rates below 40%. The average baseline completion rate was 10%. Two CWS targets were presented for each passage. One of them represented the unit of global predictions, the other the unit of local predictions. The presentation order of the two CWS targets was random.

#### Procedure

Participants read the same passages as those participants read in Experiment 1. After each passage, participants pressed a key, and the first CWS appeared on the screen for 2 s. It was then replaced by the second CWS, which also remained present for 2 s. For each CWS, the participants were instructed to say aloud the first word that came to their mind that fit the blank. The participants were told that they must complete the CWS task with a word that was coherent to the prior text. The words representing the global prediction and the local prediction were presented in a counterbalanced order. Experiment 1a and 1b are totally two separate tests on two groups of different participants, so answering CSW task in Exp 1a would not affect the performance on Exp 1b.

The experimenter recorded all the responses to the CWS targets. The CWS target word was followed by a true/false comprehension question about the passage. Each question required simple factual knowledge of the sentence. Participants’ answers were recorded, and immediate feedback was given. Participants were run individually in a single session lasting approximately 30 min.

#### Designs

There were four stimulus presentation lists. Each list contained an equal number (6) of passages from each of the four versions and all eight filler passages. A balanced Latin Square design (Kirk, 1995) ensured all 6 passages from one version were presented at different serial positions and were followed by a different reading version of the same passage only once in all four reading lists. Two filler passages were presented in the same order before and between every two material versions in each list.

This resulted in a 2 (*elaboration*: high vs. low) × 2 (*predictability*: inference vs. control) randomized block factorial design was used. Both of these factors were within-subject random factors. For all analyses we were analyzing F1, for the sake of saving space. We used F instead of F1 on the subject level data as there were no item-level research questions.

### Results

A word stem response was coded as a *target* response (or an inference-relevant response) if it had been completed with the word that was intended to be relevant to the inference. Scores were converted to percentages. The overall rate of target responses was not very high for either global or local predictions. As seen in Table 2, in the high elaboration context, the average percentage of target responses for global predictions was 12.3 out of 24 words (i.e., 51%), and for local prediction was 7.4 of 24 words (i.e., 31%). In the low elaboration context, the average percentage of target responses for global predictions was 11.7 out of 24 words (i.e., 49%), and for local predictions was 3.8 out of 24 words (i.e., 16%).

**TABLE 2:** Percentage of target words generated in CWS task.

	Prediction type			
	Local		Global	
Target sentence condition	Low elaboration	High elaboration	Low elaboration	High elaboration
Predictable	15.8%	30.8%	48.7%	51.7%
Control	13.7%	11.1%	18.7%	30.8%

*CWS, Constrained Word Stem Completion Task requires the participants to generate a word which starts with given letters.*

The mean proportions of targets completed for either global or local predictions were analyzed with a repeated-measures analysis of variance. We tested the simple effect of predictability separately for high elaboration and low elaboration (i.e., predictability vs. control for high elaboration, predictability vs. control for low elaboration). All effects reported as significant reached at least the 0.05 alpha level.

There was a significant main effect of *elaboration* on target completions for both global predictions [*F*_(1, 28)_ = 12.58, MSE = 1542.229, *p* < 0.001, η^2^=0.13] and local predictions [*F*_(1, 28)_ = 5.486, MSE = 1871.341, *p* = 0.031, η^2^=0.11]. There was a significant main effect of *predictability* on the target completion only for local predictions [*F*_(1, 28)_ = 8.72, MSE = 1245.875, *p* < 0.001, η^2^=0.21], but not for global predictions [*F*s < 1]. The *elaboration* by *predictability* interaction on target completions was found only for global predictions [*F*_(1, 28)_ = 11.84, *p* = 0.001, η^2^=0.17], not for local predictions [*F*s < 1].

### Discussion

There were three major findings in Experiment 1. First, the CWS task showed that a higher elaboration on the global aspects in the narrative resulted in a greater likelihood of both local and global predictions. Second, there was evidence of global inference activation regardless of the degree of text elaboration on the global aspects in the narrative. Third, and most crucially, the critical event was strongly predictable at the local level only when there was less elaboration. This pattern of results suggests a competition between the potentially global inferences and the local inferences. When there was less competition from the global inference during online processing, the primary local inference is more easily detected, probably it did not exit at all in the high-elaboration context. Follow-up experiments planned to continue explore this.

## Experiment 2

In Experiment 1, we found no significant interaction effect on target completion for local predictions; i.e., even as the degree of text elaboration on the global aspects in the narrative increases, the saliency of the local prediction did not decrease. However, the constrained word-stem completion task may not have been the most sensitive measure for detecting this interaction. In Experiment 2, we tested this distant contextual effect using the inferential-word probe-naming task, a more sensitive measure for online detection of inference generation.

Specifically, we tested the following hypotheses. *First*, the effect of elaboration should be reflected in a delayed online probe-word naming time if the degree of elaboration of the global aspects in the text is high. *Second*, if the text supports predictive inferences, a main effect of *predictability* should emerge in a quicker naming latency of the probe word in the inferential context relative to the control (regardless of text elaboration). *Third*, if the predictability effect is attenuated by the previous elaborated text, there should be an interaction effect of elaboration by predictability. There should be an interaction effect of elaboration by predictability.

### Materials and Methods

#### Participants

Graduate and undergraduate students enrolled in a large Southeastern U.S. university participated in this study in exchange for bonus points toward a course grade (*N* = 39). The average age of the participants was 27.3 years, ranging from 18 to 35, and 56% were female. Three participants’ data were discarded from the final analyses because their accuracy rates for comprehension questions were below 85%.

#### Materials

The passages were the same as the ones used in Experiment 1. The probe word represents the local prediction that is evoked from the predictable target sentence. These probe words have been assessed as the most likely inferential concepts (McKoon and Ratcliff, 1986; Linderholm, 2002), and their online activation has been successfully detected (Klin et al., 1999). The length of the probe words ranged from 3 to 7 letters with a mean length of 4.92 letters.

#### Designs

The design was the same as Experiment 1.

#### Apparatus

The computer on which test materials were presented to participants was equipped with a Stimulus-Response (SR) box. There were three response keys designated on the response box: The “Line-Advance” key was used to move the computer screen to the next page, and “Yes” and “No” keys were used to answer simple Yes-No comprehension questions at the end of each passage. A microphone linked to the response box monitored the participants’ naming of the probe word and triggered the voice key to record the probe word naming time. Before the experiment, the participants completed five practice trials.

#### Procedure

Each participant was run individually in a 30-min silent reading session. The participants pressed the button on the response box to advance the sentences presented on the computer screen one at a time. Each trial began with the presentation of the words PRESS THE SPACEBAR WHEN READY. The last sentence from each passage (i.e., the target line of either predictable or control sentence) was followed by a “^***^” cue, then a probe word flashed for 2 s to be named by the participants.

Participants were instructed to name the word out loud as quickly as possible. After they named the word, it was erased from the probe-naming screen on the computer. After the probe word, a simple “Y/N” comprehension question flashed for 2 s. Participants were given enough time to make a “Yes” or “No” response to the comprehension question they just read. Feedback on the answer to each comprehension question was given at the end of each passage to ensure the participants read each passage carefully. Participants were allowed to take a short pause before reading another passage.

### Results

The mean naming times are presented in Table 3. Outlying naming times were defined as those 2 standard deviations from the mean (Tukey, 1977) and discarded, resulting in loss of less than 5% of the data from all of the analyses. The amount of data eliminated did not differ significantly between conditions. All analyses were significant at the standard alpha level of 0.05, unless otherwise indicated.

**TABLE 3:** Average latency on probe word pronunciation (and SDs) in milliseconds (ms).

Predictability type				
Elaboration type	Inference	Control	Difference
High	494.16 (57.23)	533.48 (106.28)	39.32*
Low	469.23 (57.12)	526.04 (71.21)	52.77*
Difference	24.93*	7.44	–

Values enclosed in parentheses represent the standard deviations. *p < 0.05.

A 2 (elaboration: low vs. high) × 2 (predictability: predictable vs. control) Repeated Measure ANOVA was conducted on the subject mean correct naming latencies (see Table 3). Elaboration [*F*_(1, 35)_ = 5.450, MSE = 1729.695, *p* = 0.025, η^2^=0.25] had a main effect on the naming latencies such that high-elaboration conditions (514 ms) were responded to more slowly than low-elaboration conditions (498 ms). There was also a main effect of predictability [*F*_(1, 35)_ = 54.486, MSE = 1526.344, *p* < 0.001, η^2^=0.34] such that the average naming time in the Inference conditions (42 ms) was significantly faster than in the control conditions (529 ms) (*p* = 0.012). Lastly, there was a significant elaboration by predictability interaction [*F*_(1, 35)_ = 11.84, *p* = 0.001, η^2^=0.06] such that the predictability effect (elaboration vs. control) was larger for the low-elaboration condition than the high-elaboration condition.

### Discussion

The naming time data suggested a reliable *immediate priming effect* of the predictable target sentence regardless of the degree of text elaboration. Further, this effect was even larger when the elaboration on the global aspect of the narrative was low. Thus, we conclude that inferential-probe naming task shows evidence of the activation of local inferences, regardless of the degree of text elaboration.

This is not inconsistent with what we found in Experiment 1, but it reveals a more detailed picture of text comprehension. Consider the levels of processing of CWS and naming tasks. CWS represents the SI of what has been read with what is coming next in text comprehension. But the naming task reflects a more superficial level of text processing, in which readers may access the word at the phonological level only without being able to activate the semantic meaning of the inferential-probe word. These differences may affect the readers’ ability to comprehend and make inferences online.

However, an alternate explanation of this discrepancy relates to readers’ working memory and how much information they can process continuously while holding some amount of information in their text memory. This account predicts that individual differences in working-memory capacity may thus predict the degree to which readers make inferences online. To reconcile the discrepancy between two experiments, we adopt an individual-differences approach to further explore whether and to what extent the distant contextual factor influences the processing of immediate priming effect of the crucial target sentence and the maintenance of both global and local immediate text memory.

## Experiment 3A

In Experiment 3A, we again examined participants’ naming times in the same four passage conditions used in prior experiments. However, we now examined individual differences in this process. Specifically, we asked whether individuals differ in their capacity to activate local predictions upon encountering contextual elaboration on the global aspects of the narrative.

This prediction ties into broader theoretical claims about individual differences among readers. In particular, readers with lower WMC may have difficulty in processing the complex text online because they have difficulty suppressing irrelevant information (the Skilled Suppression Hypothesis; Gernsbacher and Faust, 1995). Furthermore, the degree of the readers’ activation of inference, regardless of high or low span in reading, could affect their memory for the text tested in Experiment 3B.

### Materials and Methods

#### Participants

Seventy-three college students in a large Southeastern U.S. university participated for course credit, $10 cash, or both. They were recruited from 28 academic programs from 11 fields of study in order to generalize across a variety of college reading backgrounds. Thirty-seven (51%) were female and thirty-six (49%) were male. Their ages ranged from 17 to 46 with a mean age of 25. All participants were required to speak English as their first language. Three participants made more than 25% errors on comprehension questions and were excluded from the analyses. Therefore, the data analyses were based on 70 participants.

#### Materials

The experimental passages were the same as those in Experiment 1 and 2.

#### Measures

In Experiment 3A, we used a version of Reading Span task (Daneman and Carpenter, 1980; Turner and Engle, 1989). This task is designed to require working memory storage in the face of processing (or distraction) in order to engage executive attention processes (Conway et al., 2005). The task involves reading a series of sentence-letter strings (e.g., *On warm sunny afternoons, I like to walk in the park. ? F*). Participants read each sentence aloud and are asked to verify whether it makes sense by saying “yes” (makes sense) or “no” (does not make sense) immediately after they finished reading the sentence, then to read the letter *F* aloud. At the end of the series, participants are asked to write down the sequence of letters in exactly the same order they read. Each series consists of two to five strings, and the order of string lengths is determined randomly. Individuals are tested on three series of each length (12 in total); the task takes about 15 min to complete. Reading Span scores (range: 0–42) consist of the total number of letters recalled on perfectly recalled trials. According to previous studies, the test-retest reliability ranges from 0.70 to 0.80 among adults (Conway et al., 2005), and Reading Span correlates with other working memory measures with a range of 0.4–0.6 (Conway et al., 2005). This means that these measures of the same construct, which theoretically should be related, are in fact related.

#### Procedure

Each participant completed the online naming task from Experiment 2, after which a 15-min Reading Span task was individually administrated.

#### Design

Participants were classified into high-, medium-, and low-span groups based on the tripartite split of the participants’ Reading Span percentile scores (see Table 4). Low-span readers (*n* = 22) were defined as the lowest scoring third (mean percentile score = 15.8; range = 1–29.5); medium-span readers (*n* = 23) were defined as the middle-scoring third (mean perceptual score = 46.6; range = 35.56–63.70); and the high-span readers (*n* = 26) were defined as the highest scoring third (mean percentile score = 82.9; range = 68.49–97.95).

**TABLE 4:** Percentile scores of working memory capacity measured by RSPAN.

Low *(n* = 22)		Moderate *(n* = 23)		High *(n* = 26)	
Mean	*SD*	Mean	*SD*	Mean	*SD*
15.8	5.6	46.6	5.7	82.9	7.3

A 2 (elaboration: low or high) × 2 (predictability: predictable or control) × 3 (span: low, medium, or high) repeated-measure mixed design was conducted on the naming times and the proportion of the local prediction, respectively. The former two variables were within-subject variables; the latter one was a between-subject variable.

### Results

The naming time analyses were conducted based upon the aggregated mean of each participant’s naming times in the 6 passages in the same reading condition. For each participant, there were four aggregated means representing the naming times in their four reading conditions.

Outlying naming times of more than 2 standard deviations from the mean were discarded, resulting in the loss of 4 percent of the data. The mean naming times are presented in Table 5.

**TABLE 5:** Average latency on probe word pronunciation (and SDs) in milliseconds (ms).

	High elaboration		Low elaboration	
	Predictability	Control	Predictability	Control
Low	460 (49.8)	461 (61.1)	454 (48.5)	479 (61.3)
Medium	511 (62.8)	528 (55.1)	474 (47.6)	513 (57.5)
High	503 (64.0)	525 (58.3)	468 (55.8)	511 (59.3)

Averaging over the working-memory groups, Experiment 3A replicated the findings of Experiment 2, showing a significant elaboration by predictability interaction. Naming times were faster in the predictable condition than in the control condition, and this main effect of predictability was significant [*F*_(1, 67)_ = 33.04, *p* < 0.001, η^2^=0.21]. Naming times were faster in the low-elaboration condition than in the high-elaboration condition, and this main effect of elaboration was significant as well [*F*_(1, 67)_ = 12.98, *p* = 0.001, η^2^=0.07].

Next, we turn to the effects of WMC on activation of local predictions. The ANOVA found that the 3-way interaction of Elaboration × Predictability × Span was not significant [*F*_(2, 67)_ = 0.95, *p* = 0.39, η^2^=0.07]. However, the main effect of span was marginally significant [*F*_(2, 67)_ = 2.67, *p* = 0.07, η^2^=0.19], and there was a significant Elaboration x Span interaction [*F*_(2, 67)_ = 3.97, *p* = 0.05, η^2^=0.23].

To probe this interaction further, we conducted comparison among the three span groups in the predictable condition under each context. In the low-elaboration context, the naming times did not differ across three span groups, suggesting that all three groups of readers could either activated or not activate inferences online, regardless of their WMC. But, in the high-elaboration context, the naming times among the low-span readers were significantly faster than both the medium-span readers by 52 ms (*p* = 0.01) and the high-span readers by 42 ms (*p* = 0.03). This result suggests that low-span readers did not activate the local inferences online when there is high elaboration of the global inference.

*Post-hoc* comparisons were also conducted for the mean naming times between the predictable and control conditions for three span groups using the Bonferroni criterion (*p* = 0.25), as there were 5 comparisons in total. The naming times were reliably faster in the predictable condition than the control condition for both the high-span readers [*t*(21) = 2.57, SE = 4.37, *p* = 0.02 (one-tailed)] and the medium-span readers [*t*(25) = 3.57, SE = 6.37, *p* < 0.001 (one-tailed)]. These results indicate that high- and medium-span readers—as defined by the reading span task (Unsworth and Engle, 2006)—activated local predictions during reading. For the low-span readers, however, the times did not reliably differ between the predictable and control conditions, *ts* < 1.

### Discussion

In Experiment 3A, the latency to name the inference-critical word of the local prediction (e.g., “broke”) showed that some initial on-line encoding of the local prediction occurred in all but one experimental condition (see Table 5). For example, in the low-elaboration text, even the readers with low WMC showed a sizable facilitation effect on the probe-naming time relative to the control condition. This means that all three groups of readers activated inferences online, regardless of their WMC, in order for the inference to be able to affect naming of the probe word immediately afterward.

The one condition in which facilitation was not observed was for low-span readers reading the globally highly elaborated text. Thus, only this particular combination of a text’s comprehension demands and the reader’s processing capabilities (i.e., WMC) exhausted and/or re-directed the cognitive comprehension processes such that a local prediction was no longer afforded in this condition. In comparison to the low-elaboration text, the highly elaborated text shifted the processing demands toward the global prediction, possibly at the expense of the local prediction. It appeared as if, for the low-span readers, the processing demands of the highly elaborated text did not also permit the on-line generation of the local prediction, but for the globally less-elaborated text they were still able to do so. The high- and median-span readers, on the other hand, had more processing resources, yielding an on-line generation of the local prediction, even for the globally highly elaborated text. Greater WMC thus supports a more complete processing of the text, namely the on-line generation of a local prediction in addition to the possible global inference (see the results of Experiment 2); by contrast, low-capacity readers must adjust their processing to the higher demands of the globally elaborated text and thereby abandon the generation of the local prediction.

## Experiment 3B

Experiment 3A provided intriguing evidence that additional processing demands can break the ability to generate a local prediction online. What are the consequences of this change for long-term comprehension and memory for the text?

In Experiment 3B, we now consider how local and global inferences may reappear or even be newly constructed when the same readers are later asked to recall the text. A new construction of a local (or global) prediction may even occur when there was no initial activation of it during reading because the recalled text affords the generation of the respective inference, in a similar manner as when the text was first read.

Specifically, we use a text reproduction task in which participants are asked to reproduce the text in exactly the way as they have read it and without adding anything. In this task, (re-)produced inference statements are often considered “intrusions” and classified as errors by some researchers. However, such inferences in form of local or global predictions are the product of integrating a text’s new information with a reader’s prior knowledge. Therefore, they are a signature phenomenon of successful comprehension. We will consequently abstain from the evaluative denotation “intrusions” and refer to these cognitive products more descriptively as local predictions and global predictions.

In Experiment 3B, the same participants who had already completed Experiment 3A were furthermore asked upon completion of Experiment 3A to reproduce the texts which they had just read immediately afterward. Experiment 3B thus allowed not only us to determine how many local and global inferences were stated in the participants’ text reproductions, but to assess their relation to the magnitude of the priming effect for naming the target word related to the local prediction. In other words, do conditions that show the largest priming effects also show the most inferential text memory in the reproduction?

### Materials and Methods

#### Participants

All participants of Experiment 3A, consisting of 22 low-, 23 medium-, and 25 high-span readers, were asked to reproduce the texts which they had just read.

#### Procedure

Each participant had already completed the online reading session and the Reading Span task (15 min) before they were provided with a cued-recall booklet to recall whatever they could remember from the passages they had read. The first sentence of each of the 24 passages was given in the booklet. Sufficient time was provided so that all participants could complete these tasks in an orderly fashion.

### Results and Discussion

Table 6 shows a selected example of the participants’ text reproductions for each of the six experimental conditions. As expected, these examples show that the participants’ reproductions reflect not verbatim memory of text they had read, but rather its meaning. It is easily seen that, in comparison to the studied text, some propositions from the text have been omitted while others have been added. Similarly, depending on the particular reader and the particular text, the gist of text, as well as the temporal and causal structures of the described events, may or may not be present in a given text reproduction.

**TABLE 6:** Sample recall text in low and high elaboration condition among three WMC groups.

WMC	High elaboration	Low elaboration
Low	Steven had a terrible temper. Susan said that if one more act of violence was committed in the house, she would leave. Steven got a new job at Sears. Susan left the kitchen in a huge mess.	They decided to split up because Steven was abusive. They decided to move to change the scenery. One day, Susan had not cleaned up the apartment. Steven got very angry. He picked up a vase and threw it.
Medium	After many years, Steven became abusive. Susan joined a battered women’s group. Susan warned Steven that if he displayed the slightest bit of violence, she would leave him. Steven couldn’t bare the thought of Susan leaving and felt his life would be over if she did. One day, Steven found a mess in the kitchen and couldn’t control his anger. Steven threw a small vase across the room.	Steven had an anger problem. Susan said that she would leave Steven if he had one more incident. Steven just got a new job at Sears as a manager. Steven had long hours and lots of stress. Steven and Susan had difficulty adjusting to the new hours. One day, Susan left dirty dishes in the sink. Steven got very angry and threw a vase at the wall. The vase crashed against the wall and when Susan got home, she was very angry and left.
High	Steven abused Susan until she eventually had enough. Susan told Steve that she would leave him if even a tiny violent event occurred in the house. Susan left the kitchen a mess 1 day. Steven tried to deal with it, but got really angry. He threw a small vase against the wall and broke it. Finally, Susan saw the broken vase and left Steven.	Susan was tired of an abusive relationship. Steve was told that if one more violent burst were to happen, she would leave him. Steven was recently appointed assistant manager at Sears. This meant he had more responsibility and more stress. One day, Steven came home from work. Having noticed Susan had left a mess, Steve was enraged and threw a porcelain vase against the wall. The porcelain vase will break when thrown.

To assess the individual text reproductions in a more detailed manner, each reproduction was assessed according to five attributes, each of which was scored as a 0 (not fulfilled) or a 1 (fulfilled). The five attributes were: (a) whether the reproduction included the inference-related target word for the *local* prediction, (b) whether the reproduction included the inference-related target word for the *global* prediction, (c) whether the reproduction presented the gist of the text (which was invariant across the different versions of each experimental text) (d) whether the temporal structure of events was reproduced, and (c) whether the causal structure of the events was reproduced. For the particular example reproductions shown in Table 6, the respective scores can be seen in Table 7.

**TABLE 7:** Two samples of text recall scores among three WMC groups in two types of texts.

Text characteristics	Working memory	Local prediction	Global prediction	Gist	Temporal structure	Causal structure
Low elaboration	Low	0	0	0	0	0
	Medium	0	1	1	1	1
	High	1	0	0	1	1
High elaboration	Low	0	1	1	0	0
	Medium	0	1	1	0	1
	High	1	1	1	1	1

To assess the reliability of this coding procedure, two persons coded the recall data. The first author coded all of the data on key ideas of the exact recall of the primary local prediction (e.g., Susan’s leaving), and five subcategories of the text coherence including local and global predictions, and gist, temporal structure and causal structure of the text. Meanwhile, a graduate research assistant analyzed 1/8 of the participants’ responses. Both coders were blind to the experimental condition in which the reproduction occurred. The Pearson correlation coefficient between the commonly coded scores of two raters on each categories was used to assess interrater reliability because it considered the base rates of these categories. The interrater reliability coefficient ranged from 0.71 to 0.86 for the five coded variables (see Table 8), indicating acceptable reliability.

**TABLE 8:** Inter-rater reliability for coding of accurate recall and text coherence (*n* = 216).

Research questions	Coding scheme		Category	Percentage of agreement
Question 3	I. Exact recall		Primary local prediction	92.8%
Question 4	II. Text coherence	Local	1. Local prediction	92.3%
			2. Global prediction	96.2%
		Global	3. Gist	95.8%
			4. Temporal structure	91.8%
			5. Causal structure	96.9%

In addition to this scoring of the text recall, an immediate priming effect (IPE) for the local prediction was calculated from the data obtained in Experiment 3A by subtracting the mean naming times of the probe words in the predictable conditions from those in the control conditions. A positive difference score indicates that participants named the probe words more quickly in the predictable condition than the control condition, suggesting that local predictions were activated online (Murray and Burke, 2003), and was scored as a 1. A negative or no difference score was scored as a 0. We calculated this priming effect for local predictions separately for the low elaboration context (IPE-L) and the high elaboration context (IPE-H).

### Working Memory Capacity Effect on (Re-)producing a Predictive Text

To examine reproduction of the inferences, only the low-elaboration inference and high-elaboration inference conditions were relevant because only these two conditions were designed to elicit predictions (whether local or global); no predictions were expected in the control conditions.

A 2 (elaboration: low or. high) × 2 (aspect: local or global) × 3 (WMC: low, medium, or high) repeated measure ANOVAs was conducted on the (re-)production rates of the local prediction in these two conditions. All effects reported are significant at *p* < 0.05.

There were significant main effects of elaboration [*F*_(1, 67)_ = 6.28, *p* = 0.016, η^2^=0.21] and aspect [*F*_(1, 67)_ = 18.90, *p* < 0.001, η^2^=0.16], but no significant span effect [*F*_(2, 67)_ = 0.43, *p* = 0.515, η*^2^*=0.11]. High elaboration context were recalled better than the low elaboration context. There was a significant Elaboration × Aspect interaction effect on the text recall [*F*_(1, 67)_ = 4.95, *p* = 0.031, η^2^=0.23], and a significant Elaboration × Aspect × Span 3-way interaction effect[*F*_(2, 67)_ = 4.03, *p* < 0.06, η^2^=0.11], but there was no Aspect × Span interaction [*F*_(2, 67)_ = 1.37, *p* = 0.248, η^2^=0.10] To break down the Elaboration × Aspect × Span interaction, we performed planned contrasts comparing the (re-)production rates for each span level (high vs. low span) in each of the two types of text: high and low elaboration texts.

### Text Recall for the High Elaboration Text

The high elaboration text had more sentences supporting the global inferences than sentences supporting the local inference. Therefore, it is expected that readers would include more global than local inferences in their text reproductions. As can be seen from Table 8, this prediction was supported for all three working memory groups. However, there was an overall difference among the groups such that high-span readers (re-)produced the inferences more frequently than low-span readers; this was true for both global inferences (60% vs. 54%) [*t*(46) = 4.47, *p* = 0.04] and inferences (45% vs. 33%) [*t*(46) = 5.92, *p* = 0.02].

For this high elaboration text, the high-span readers also showed more structure in the recalled texts than the low-span readers. Specifically, high span readers reproduced the temporal order more frequently (37.9%) than low span readers (23.0%) [*t*(46) = 6.11, *p* = 0.01], as well as the causal structure (22.3% vs. 8.8%) [*t*(46) = 7.32, *p* < 0.001]. The scores of the medium span readers were in between in both cases. The scores for the gist of the text reproductions showed approximately the same pattern.

It is interesting to note that the online activation of the local inference increased with a reader’s WMC, just as did the percentages of local inferences and the percentages of global inferences in text recall. The results of a hierarchical regression analysis (Guan, 2007), which are partially reproduced in Table 9, show, for both local and global inferences, that the online priming effect is the best predictor for whether inferences become included in the text reproduction.

**TABLE 9:** Means and standard deviations of coherence ratings in low and high elaboration reading conditions, low- and high-span groups when local predictions were activated (*n* = 70).

Text characteristics	Reader characteristic	Reading online measures	Assessed quality of recalled text in terms of									
			
Elaboration	Working memory	Priming effect	Local prediction	*SD*	Global prediction	*SD*	Gist	*SD*	Temporal	*SD*	Causal	*SD*
Low	Low	25.35	44.1	27.2	52.7	31.1	38.4	26.5	18.0	21.1	16.2	11.3
	Medium	39.12	33.8	24.1	36.7	20.8	35.0	23.0	19.2	17.8	11.9	8.9
	High	42.47	31.7	23.4	33.1	20.4	24.2	21.0	13.3	13.6	18.8	15.1
High	Low	1.38	33.4	24.1	53.7	26.5	33.4	26.7	23.0	18.3	8.4	8.8
	Medium	16.42	42.0	22.2	58.8	25.6	46.7	23.1	35.5	14.5	23.3	10.5
	High	22.79	44.8	25.1	60.0	20.7	44.4	24.7	37.9	28.8	21.0	22.3

At least for high-elaboration texts, then, the online priming effect for the local prediction accounts for more variance than the reader’s WMC. Further, because the online inference activation score in turn predicts the (re-produced) local and global inferences, this score can be viewed not only a measure of local inference, but a predictor for the overall quality of comprehension, as measured by text recall.

### Text Recall for the Low Elaboration Text

We next turn to the low-elaboration text. Because this text is less demanding for a reader, the respective results may turn out quite differently. Indeed, for the low-elaboration text, the relation between the on-line local inference generation and the (re-)produced local and global predictions was quite different. Here, a smaller amount of immediate priming (as typically seen for the low-span readers) was associated with a *high*er percentage of (re-)produced local and global predictions in text recall, and a higher amount of immediate priming (typically, the high-span readers) was associated with a lower percentage of (re-)produced local and global predictions in text recall.

This starkly different pattern of results between the low- and high-elaboration texts can be explained by the different processing demands of the two texts. Because the highly elaborated text is longer, it is more time-consuming to comprehend, and it requires more working memory. Readers therefore may abandon a strategy of trying to reproduce the text verbatim and instead focused more on the meaning and the gist of the text. Consequently, better comprehenders (as measured by the online priming effect) would have obtained a better gist representation, which naturally included both the global and local predictions.

By contrast, because the low-elaboration text is shorter, the readers may have been able to focus on the instruction to reproduce the text verbatim. Thus, the better comprehenders (as measured by the on-line priming effect) were also better able to exclude the global and local predictions from their text recalls, yielding a negative correlation between online priming effect and (re-)produced inferences (see Table 10).

**TABLE 10:** Summary of beta weights results from final step of hierarchical regression analyses predicting text recall from working memory and the immediate priming effect (*n* = 70).

Level of text elaboration	Predictor variables	Local inference	Global inference
Low	IPE-L	0.12*	0.45***
	WM	−0.27	−0.36**
High	IPE-H	0.47***	0.37**
	WM	0.24	0.14*

*IPE, Immediate Priming Effect; WM, Working Memory Group; (−), indicates the regression weight is negative.*

*p < 0.05, **p < 0.01, and ***p < 0.001 (2-tailed).

## General Discussion

In a series of experiments, we examined the effects of elaboration and predictability on the predictive inference activation and its maintenance in Experiment 1 and 2. We also examined how WMC could possibly impact this moment-to-moment inference activation online in Experiment 3a. Lastly, we considered the coherence of later offline text memory in Experiment 3b. The contribution of Experiment 1 and 2 were to produce replicable results by using different detecting tasks, so as to strengthen the argument that the predictive inference could be made online, but only when the contextual conditions could support its online activation. The central theme of the study aimed to explore the offline text memory when the online inference were detected, and how this online activation of inference could facilitate the resonance of offline text memory.

There were three notable findings. First, elaboration increases the likelihood of both local and global predictions (Experiment 1), and it slowed the time course of predictive inference activation (Experiment 2). Second, local predictions demonstrated that the immediate priming effect reflected deep text processing to facilitate a more unified text memory, and high span readers benefited more from this effect (Experiment 3A and 3B). Third, the high-span readers achieved a more coherent integration between the multiple inferences and other parts of text related to the inference on the situational level (Experiment 3B). In the remainder of the discussion, we focus our theoretical exploration on the implications of the current study for attentional competition vs. SI accounts of the linkage from online activation to coherent text memory.

### Low-Span Readers Had Trouble in Intertwining Both Local and Global Inferences

Our most important finding was that high-span readers processed the implicit and knowledge-based inferential concept differently from low-span readers. Specifically, high-span readers were sensitive to multiple possible inferences that could be drawn. In contrast, the low-span readers were only sensitive to the inferential event in immediate active memory, and they had trouble in making both local and global inferences coherently intertwined with each other.

This general pattern of results was consistent across experiments and was not affected by the task that was associated with reading. This indicates that, when comprehending the text, high-span readers can generate a larger workspace and thereby achieve a SI which encompasses the local and global predictions. Low-span readers have a smaller workspace. For a highly elaborated text, the demands of text integration are larger than these low-span readers’ available workspace. Consequently, an attentional split occurs, which drops the local aspects of the processed text (including the possible local inference) from the workspace and thus from processing and from the SI processes that achieve comprehension. The low-working memory participants therefore develop a more coarse-grained representation, which does not encompass a detailed event structure.

### Low-Span Readers Have a More Restricted Working Memory to Block the Immediate Text Memory

The effect of working memory on predictive inferences has been examined in previous studies; however, those studies focused on which properties of the text were causally sufficient to produce predictive inference. These studied concluded only high-span readers make predictive inferences if the context is sufficient (Calvo, 2000; Cook et al., 2001; Linderholm, 2002). The result of this study suggested that low-span participants were not affected by the distant information. Indeed, De Neys et al. (2003) found similar results suggesting that low-span participants, due to their low WMC, could block distracting or distant information originally stored in their prior knowledge. This finding is also consistent with another study (Goel et al., 2003) finding that activating the most recent information at the current restricted WMC reflects an elaborated information might be blocking the given/new information in the upcoming text.

According to the more general working-memory literature, working memory functioning is closely related to inhibition (e.g., May et al., 1999). Taken together, in our current study design, the low-spans have restricted working memory resources, so they failed to block the prior knowledge and therefore have little chance of making global predictions online. Therefore, it seems that in the current study, when the low-span readers activated the local prediction in their immediate text memory in a more limited manner, they were inhibiting the other distant information. Accessing one piece of information was easier and quicker than accessing multiple pieces of information. One might interpret this finding from the perspective of cognitive load. Low-span readers have a restricted amount of cognitive load in processing information. In other words, when a passage contains a degree of high elaboration that supports global prediction *and* a target sentence supporting a predictive inference, the low-span readers did not have sufficient resources to integrate all this information.

Longer naming times among low-span readers concord with findings from other investigators, who have shown that poor readers experience difficulty with word naming due to poor-quality lexical representations (Lemoine et al., 1993) and difficulty using context to integrate a newly read word (Perfetti et al., 2008). Poor readers fail to activate topic-related inferences even when inference probes were presented with a stimulus onset asynchrony (SOA; i.e., the relative timing of the onsets) condition of 1,000 ms, which allows ample time to draw an inference (Long et al., 1994). This pattern could be interpreted in terms of the Skilled Suppression Hypothesis, such that weak inference on the word-level activation could be due to the skilled suppression of irrelevant associations among the good readers, but the quick enhancement of appropriate associations among the unskilled readers (Gernsbacher and Faust, 1991). Similar findings have been reported from a word recognition task in a study on syntactic ambiguity (Pearlmutter and MacDonald, 1995) and from reading predictable sentences (Perfetti and Roth, 1981). Both of these studies suggested that poor readers had quicker word recognition speeds on the target sentence because they were not actually processing the sentence at a deeper level.

Similarly, as revealed by the Experiment 1 and 2, when reading the high-elaboration text, high-span readers integrated the different text events in an all-encompassing and coherent manner at the global level. But low-span readers, on the other hand, had fewer processing resources available, which they allocated for successful comprehension at the global level while abandoning the details (i.e., the generation of the local predictions). It is quite interesting that, despite their limited processing results, the low-span readers nevertheless allocated these resources in an optimal manner by focusing on the global level, which is indeed the target level of successful comprehension. Thus, both the high- and low-span readers can be seen as optimally using whatever memory resources they have. High-span readers adopt an immediate integration mechanism in reading, which allows them to integrate their current processing with the earlier memory of text for comprehension. Low-span readers, by contrast, adopt a skilled attention competition mechanism in reading; they suppress the local details while focusing attention on the global idea of the text for successful comprehension.

A final important finding of this study is that the magnitude of the online local prediction effect is a very good measure of a reader’s functional working memory in text comprehension and an overall measure for comprehension success. Why do working memory and text comprehension relate in this way? When people are making inferences online, they are engaged in a deep level of text comprehension. This deep comprehension involves integration and construction during reading, requiring readers to hold the prior text memory in mind and integrate the upcoming information in order to construct a situation model of the text (Kintsch, 1994). These processes are exactly those that require working memory to hold the background contextual information, predict what will happen next at the local level, and merge them into a global understanding of the overall text.

## Conclusion

The take-home message of this current study is threefold. First, the probability of making inferences in online reading is a function of multiple factors, including text elaboration and predictability of the text as intraindividual differences, and readers’ WMC as an interindividual difference factor. Second, the degree to which the comprehenders adopt an attentional-competition vs. semantic-integration mechanism in deep text processing—as represented by inference-drawing—is reflected by readers’ engagement of reading. If local inference-making can occur online, the chance of text integration is higher. This usually happens for the low-span readers. But high-span readers can split their attention between the local and global text; therefore, they have a greater ability to make global inferences and are able to use the top-level topic of the text. Finally, local inferences, when they occur, are indicator of deep text processing that can enhance memory for the global aspects (gist, causal structure and temporal sequence) of the text. Therefore, we concluded that the online activation of the inference, when supported by the contextual elaboration, would facilitate the online memory in the long run (Ericsson and Kintsch, 1995; Guan, 2007).

## Data Availability Statement

Publicly available datasets were analyzed in this study. This data can be found here: https://pan.baidu.com/s/1jn7MX2RLp_Rl03ZQq6bUJA, retrieval password: 1234.

## Ethics Statement

The studies involving human participants were reviewed and approved by the Florida State University. The patients/participants provided their written informed consent to participate in this study.

## Author Contributions

CG and BM designed the study. CG, MG, and CZ conducted the research. CG analyzed the data. CG and SF wrote the manuscript. All authors contributed to the article and approved the submitted version.

## Conflict of Interest

The authors declare that the research was conducted in the absence of any commercial or financial relationships that could be construed as a potential conflict of interest.

## Publisher’s Note

All claims expressed in this article are solely those of the authors and do not necessarily represent those of their affiliated organizations, or those of the publisher, the editors and the reviewers. Any product that may be evaluated in this article, or claim that may be made by its manufacturer, is not guaranteed or endorsed by the publisher.
